# Circularity in waste management: a research proposal to achieve the 2030 Agenda

**DOI:** 10.1007/s12063-023-00373-0

**Published:** 2023-04-21

**Authors:** Rocío González-Sánchez, Sara Alonso-Muñoz, María Sonia Medina-Salgado

**Affiliations:** Department of Business Administration (ADO), Applied Economics II and Fundaments of Economic Analysis, Rey-Juan-Carlos University, Madrid, Spain

**Keywords:** Circular economy, Waste management, Agenda 2030, Sustainable Development Goals, Research proporsal, Bio- and eco-friendly models

## Abstract

Waste management is the main challenge in the transition away from the linear "take-make-dispose" economy. Incorporating the principles of circularity in waste management would facilitate the achievement of Sustainable Development Goals. This paper aims to provide state-of-the-art research about circular waste management in the fulfillment of the 2030 Agenda. For this purpose, bibliometric analysis by VOSviewer and SciMat software is used to define the evolution and to detect research trends. Based on the main gaps identified in studies, a research agenda to guide for further opportunities in this field is suggested. The results obtained four clusters that address sustainable industrial infrastructure, biological waste management, recycling in developing countries and recovery processes. Four research propositions are established, focusing on plastic waste management and generation trends, circular municipal waste management, more sustainable landfill management, and enablers such as indicators and legislation. The transformation towards more bio and ecological models requires social, regulatory and organizational tools that consider the best interests and capacity of companies, public authorities and consumers. In addition, policy implications are considered.

## Introduction

Circular economy (CE) is a regenerative and restorative system, which allows the conservation of the value of raw materials by breaking with the concept of end-of-life of products, minimizing waste and emissions and increasing efficiency, through recycling, reusing, and remanufacturing, among others (Ellen MacArthur Foundation 2017). This paradigm represents a further step towards sustainability supported by its three fundamental pillars—economic, environmental and social sustainability (Muñoz-Torres et al. 2018). The circular system is based on the principle of material balance, seeking regeneration of natural systems, which implies the minimisation of waste and pollution. In this way, changes already begin to emerge in the design phase (Foschi et al. 2021) and go beyond the production system, reaching the development of new patterns of consumption and use by maintaining or reusing products and materials (Vanapalli et al. 2021). From an environmental economics point of view, it implies that all material or waste streams must be considered (Andersen 2007). Products have a longer lifetime, new applications and are reintroduced into the production system, closing the loop. The social aspect is fundamental to this, and coordination and cooperation with suppliers and customers must be facilitated (Martín Martín et al. 2022). In addition, making this new paradigm shift requires a new behavioural and cultural framework.

Waste management involves the transportation, collection, processing, disposal or recycling of waste materials, originating from industries, manufacturing processes and municipal solid waste. This process or system presents one of the main challenges in the transition towards circular business models (Smol et al. 2020). CE involves a waste management system that combines changes in the entire supply chain (Johansen et al. 2022), from designers and choice of materials to operators and recycling issues (Salmenpera et al. 2021).

Circular waste management comprises both the reduction in the generation of residual and household waste, but also the reintroduction of these wastes back into the production system. This reduction is achieved through the eco-design of products, by reducing waste generated in transport, by conserving material value through recycling and by achieving a longer lifetime of products (Salmenpera et al. 2021). Once the waste has been generated, it must be incorporated into the production system from the CE, either by using parts or as a source of energy through the reintroduction of biological waste, thus closing the material flow cycle (Zeller et al. 2019).

Although interest in waste management research, applying the principles of circularity, is growing, it is necessary to know state-of-the-art research trends in this area. Previous bibliometric or analytical method studies have analysed the relationship between “circularity and “waste” or “waste management” but from a different perspective to the research conducted. Recent studies have provided a qualitative view of the relationship but from very specific aspects -considering a type of waste, a geographical area or time period or one of the dimensions of sustainability-. Some research focuses on one type of waste such as Tsai et al. (2020) who analyse the link between municipal solid waste and the circular economy or Sundar et al. (2023) who examine e-waste. Ranjbari et al. (2021) examines the application of circularity in waste management, including the “closed loop” concept, up to 2020. Circular economy and closed-loop material cycles are deeply connected; however, the concept of closed-loop material cycles arose with the beginning of industrialization (Kara et al. 2022). Negrete‑Cardoso et al. (2022) considers “circular economy” to be related to “waste” and its impact on the post-Covid period. Chioatto and Sospiro (2023) discuss European economic policy issues that have promoted waste management from a circularity perspective. From a systematic literature review approach Di Vaio et al. (2023) analyse the accountability and management accounting practices of waste management related to the circular economy.

Our study presents three differentiating contributions with respect to previous studies. Firstly, we focused specifically on “circular economy” and “waste management” from a holistic perspective considering environmental, economic and social aspects. Secondly, by considering the year 2021 in the period under study, this includes one of the years with the most research on the effect of COVID-19 on waste management. The unprecedented increase of waste generated by this pandemic requires further research to enable the construction of a comprehensive circular economy model (Ranjbari et al. 2023). Thirdly, we established a relationship between our results and their contribution to the fulfilment of the 2030 Agenda. Although previous work has recognised the contribution of circular waste management to the 2030 Agenda (Di Vaio et al. 2023), a full analysis of the contribution of research by specific targets has not been carried out. Further than considering the main topics of the 2030 Agenda in the different clusters obtained, this paper establishes the relationship between the Sustainable Global Goals (SDGs) associated with waste management and the different research streams found.

The purpose of this study is to provide state-of-the-art research on the relationship between circular economy and waste management. This bibliometric analysis examines the historical evolution of research and identifies trending themes to uncover the conceptual building blocks of this field. Moreover, is setting out a research agenda about future opportunities for practitioners, policymakers, and researchers. This paper contributes to filling the existing gap on scientific literature for guiding research in the implementation of circular waste management, which is fundamental to achieving the goals outlined in the 2030 Agenda. Hence, considering the current scientific literature, we propose the following research questions:

- RQ1. How does the scientific literature structure on waste management and circular economy align with the 2030 Agenda?
- RQ2. What are the central topics and patterns within this research field?
- RQ3. What are the main research trend topics in the domain?
- RQ4. What is the research proposal on the relationship between circular waste management and the 2030 Agenda?

The paper is divided as follows: following the introduction, the literature overiew on waste management and 2030 Agenda is covered, then the methodology section is presented, describing the different phases of the process. The bibliometric results are exposed as productivity measures, considering the historical evolution of documents published in the field of waste management and circular economy and the most representative journals by authors sorted by institution, country, number of documents published and total citations. Through co-occurrence analysis, using VOSviewer software and SciMat software which displays strategic diagrams and clusters with the main motor, research topic trends in the field were identified whether basic, emerging or disappearing, and developed or isolated themes. Finally, discussions and conclusions within a research agenda are presented.

## Waste management and Sustainable Development Goals

Waste generation has increased significantly in recent years in relation to consumer patterns, activities and lifestyles. Therefore, waste management is of great environmental value (Martín Martín et al. 2022). Inappropriate waste generation has negative environmental, social and economic impacts in terms of damage to biodiversity and pollution, human health problems and the costs involved, respectively. Coping with the costs of environmental and social impacts must be considered worse than developing new and more efficient waste management systems (Sharma et al. 2021). To reduce these negative effects, the introduction of sustainable and circular issues to manage waste generation, and the collection of waste throughout the life cycle of products is required (Tsai et al. 2021). This need has been accentuated by recent crises in areas such as health, safety and energy during 2021 and 2022 (Vanapalli et al. 2021; Gatto 2022; Mišík 2022). However, these adverse historical events provide an opportunity for reflection, forcing governments and businesses to promote long overdue energy and ecological transition policies and practices (Gatto 2022; Mišík 2022). Given the need to consolidate this trend, the implementation of circularity enhances sustainability and requires a new vision in waste management (Minoja and Romano 2021).

In 2015 the United Nations adopted Agenda 2030 as a roadmap to achieving higher levels of sustainability, striving towards satisfying its 17 Sustainable Development Goals (SDGs) with the commitment of public actors, industry and society (Schulze et al. 2022). Several theories have been used in the literature to analyse these SDGs. Resource-based theory regarding natural resources is widely studied to examine waste practices that protect the environment (Agyabeng-Mensah et al. 2021). Due to the environmental impacts, some of the theories focus on pro-environmental attitudes and behaviour, such as social-practice theory (Munir 2022) and the theory of planned behaviour (Goh and Jie 2019). Regarding the association between SDGs and supply chains, a redesign towards sustainable practices is required. Transactions and economics theory have highlighted the need for changes to the decision-making process during production cycle stages to achieve sustainability goals. In addition, stakeholder and agency theories enable the achievement of SDGs, since both the collaboration and the alignment of interests in fulfilling the 2030 Agenda are required (Agrawal et al. 2022).

The relationship between waste management and the 2030 Agenda is closely linked, as it affects many SDGs. It is therefore essential that this relationship be studied. According to SDG 2, the listed items of: ‘end hunger, achieve food security, improved nutrition and promote sustainable agriculture’ require, among other factors, the minimisation of food loss and food waste to achieve efficient and sustainable agricultural production. Similarly, factors such as increasing food availability or achieving more resilient food systems would facilitate this goal (Wieben 2016). SDG 3, ‘Ensure healthy lives and promote well-being for all at all ages’, in order to reduce illness linked to water, pollution and hazardous chemicals by means of smart waste management (Fatimah et al. 2020). SDG 6 ‘ensure access to water and sanitation for all’ aims to reduce the percentage of untreated wastewater and increase recycling and reuse (Tortajada 2020). SDG 7 ‘ensure access to affordable, reliable, sustainable and modern energy’ proposes increasing the use of renewable energy and facilitating access to research on clean energy, including renewable sources (Taifouris and Martín 2023). SDG 9 ‘build resilient infrastructure, promote sustainable industrialisation and foster innovation’ advocates for the modernisation and conversion of industries towards cleaner and more sustainable models as they are required to use resources more efficiently and rationally (Dantas et al. 2021). SDG 11 ‘make cities and human settlements inclusive, safe, resilient and sustainable’ focuses on building more sustainable cities, with particular attention to air quality and municipal and other waste management. This also implies resource efficiency and waste generation-collection services (Sharma et al. 2021). SDG 12, ‘ensure sustainable consumption and production patterns’ seeks to achieve the sustainable management and efficient use of natural resources. This goal emphasises the importance of reducing different types of waste throughout the life cycle of a product or service through prevention, reduction, recycling and reuse activities (Principato et al. 2019). With regard to agro-food waste, a reduction of both food losses and food waste in the production and supply chains is proposed. SDG 13, ‘take urgent action to combat climate change and its impacts’, can affect waste treatments relevant to their environmental impact through using greener and cleaner technologies, such as anaerobic digestion (Kakadellis et al. 2021). SDG 14, ‘conserve and sustainably use the oceans, seas and marine resources’ is also linked to plastic waste management, according to marine pollution minimisation. SDG 15, ‘sustainably manage forests, combat desertification, halt and reverse land degradation, halt biodiversity loss’ can be mitigated by protection and restoration, avoiding landfill waste. Finally, SDG 17 ‘revitalise the global partnership for sustainable development’, can be enhanced owing to waste treatment development, enabled by new treatments technologies (Sharma et al. 2021).

SDGs achievement is a priority and takes on even greater importance considering the fact that eight years prior to the deadline set in the 2030 Agenda, some reports show that we are still far from meeting most of the goals. The Food and Agriculture Organisation (FAO) estimates that around 35% of employment is a direct result of food systems and the promotion and implementation of sustainable practices in the food system -including food waste and loss- which is still low, referring to unfulfilled SDG 2 (Torero 2020). Uncollected waste is one of the major issues. In terms of municipal solid waste management, proper collection is key, as mismanagement of these services can lead to dumping into waters, which directly affects SDG 6 achievement (Sharma et al. 2021). To enable both sustainable energy and industrialisation a transition towards the use of renewable and cleaner energy is necessary. Waste can be adopted as an energy resource, such as biomass waste and pyrolysis (Moya et al. 2017). However, fossil fuels are still strongly present in several industries, which negatively impact on SDG 7, 9 and 11. Waste management systems’ disruptions in relation to current situations -COVID-19 pandemic and supply crisis- have minimised recovery and recycling activity. For instance, the plastic waste proliferation caused by the pandemic resulted in both water and air pollution, due to poor and non-effective waste management. Thus, SDG 12, 13 and 14 premises are failing (Sharma et al. 2021). This also adversely affects halting biodiversity loss and the land degradation (SDG 15). In addition, there are advances in waste treatment thanks to new technologies which are starting to be implemented. For instance, anaerobic digestion and waste-to-energy technologies (Moya et al. 2017), but their application is still scarce, not satisfying SDG 17. Consequently, there is an urgent need to take additional measures to facilitate the implementation of the various sustainable measures included in the plan.

## Methodology

**Fig. 1:**
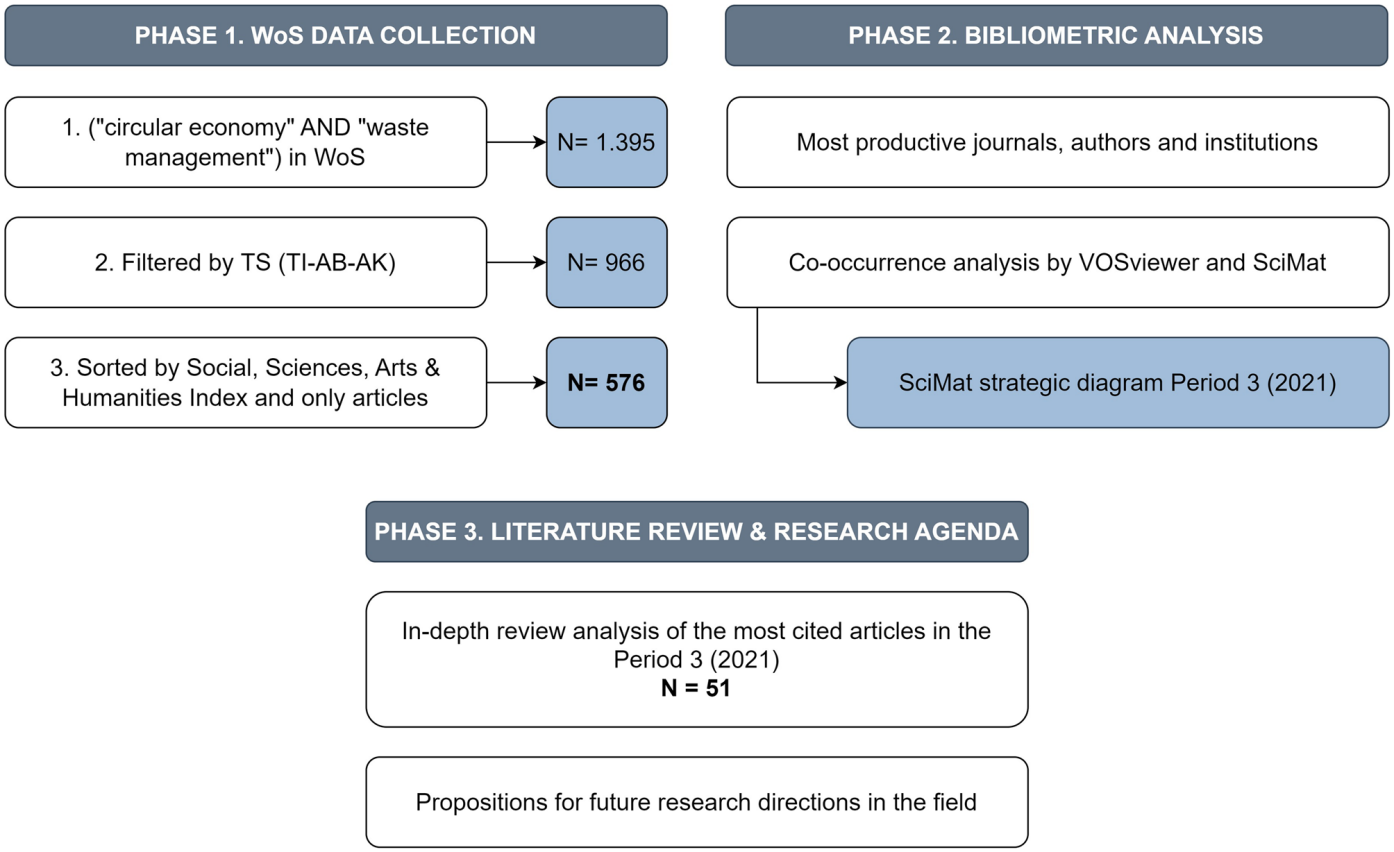
Methodological process

This study combines a bibliometric analysis carried out by VOSviewer and SciMat software, and an in-depth literature review of the articles published during the year 2021. Figure 1 shows the phases of this work: Phase 1) data collection, phase 2) bibliometric analysis, and phase 3) systematic literature review and research agenda.

### Data collection

In the first phase, documents from the Web of Science Core Collection database were collected from the period 2009 up to September 2021. The keywords used were ‘circular economy’ and ‘waste management’. This generated a total of 1.395 papers. Then, it was selected articles by topic, which includes title, abstract and authors’ keywords. retrieving 966 documents. Thereafter, we sorted the data into groups of Social Sciences Citation Index, Science Citation Index Expanded, Arts and Humanities Citation Index, taking only articles into consideration, reaching a total sample of 576 articles that were extracted and including in this analysis after a double checked in order to eliminate inconsistences.

### Bibliometric analysis

Bibliometric methodology identifies research trends providing the knowledge structure about a specific field. By examining recent published articles, network analysis shows emerging fields (Hettiarachchi et al. 2022). In the second phase, bibliometric approach was performed using VOSviewer and SciMat software to understand the latest trends in the fields of waste management and circular economy. VOSviewer is more visual and allows for the examination of co-occurrence, analysis of authors, institutions and countries (Van Eck and Waltman 2010). In this paper, SciMat completes VOSviewer analysis since it carries out the co-occurrence analysis in time periods and the evolution of these periods can be seen on an evolution map. Additionally, SciMat illustrates strategic diagrams which uncover the main research themes (Cobo et al. 2012). Furthermore, it allows one to observe the clusters of each keyword, making the analysis more complete and comprehensive.

Following on from this, VOSviewer conducts a citation analysis of the most representative journals and the most prolific authors and from here, a co-occurrence analysis is displayed. Via the SciMat tool a co-word analysis is also developed, displaying the strategic diagrams and clusters with relevant keywords, divided up into three periods according to the number of documents published, years 2009–2019 (Period 1), 2020 (Period 2) and 2021 (Period 3).

In the third and last phase, a literature review of the articles related to circular economy and waste management is carried out, in accordance with 51 documents from the motor themes of the SciMat analysis in the third period, during the year 2021, to determine the latest trends and research in the field. Finally, a research agenda is exposed regarding trending topics analysed in this work.

## Bibliometric results and productivity measures

**Fig. 2:**
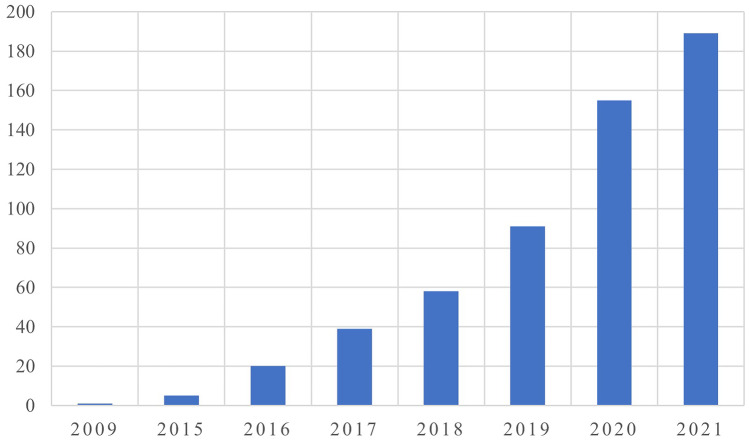
Historical evolution of publications in the field of waste management and circular economy

Figure 2 shows the historical evolution of documents published in the field of waste management and circular economy from 2009 to September 2021, considering a total sample of 576 articles. Waste management towards circularity is gaining momentum in academia according to the number of documents published in the field since 2015, coinciding with ‘The 2030 Agenda for Sustainable Development’ (United Nations 2015). In addition, other European strategies and legislative challenges took place, such as ‘Communication on closing the loop. An EU action plan for the Circular Economy’ (European Commission 2015) and ‘Communication on a monitoring framework for the Circular Economy’ (European Commission 2018) considering waste management as one of the main challenges in the transition to circular business models.

Table 1 shows the ten most representative journals sorted by number of total documents published and citations. These journals represent 60,25% of the total sample formed by 132 sources. The Journal of Cleaner Production is the most influential with 79 articles published in the field of circular economy and waste management, and a total of 1.343 cites. It should be noted that almost all sources belong to the "environmental sciences" category. None of the most cited journals belong to the social sciences.

**Table 1:** Most representative journals and authors’ institution and countries sorted by number of documents and total number of citations

**Journal**	**JCR** (2022)	**Category**	**Documents**		**TC**	**Author**	**Country**	**Institution**	**Documents**		**TC**	**h-index**
			N	%					N	%		
Journal of Cleaner Production	11.072 Q1 (24/279)	Environmental Sciences	79	13,72%	1343	Ferronato, N	Italy	University of Insubria	9	1,56%	129	11
Sustainability	3.889 Q2 (57/127)	Environmental Studies	77	13,37%	445	Torretta, V	Italy	University of Insubria	8	1,39%	129	31
Waste Management	8.816 Q1 (36/279)	Environmental Sciences	50	8,68%	658	Somplak, R	Czech Republic	BRNO University of Technology	8	1,39%	33	12
Resources, Conservation and Recycling	13.716 Q1 (12/279)	Environmental Sciences	43	7,47%	862	Smol, M	Poland	AGH University of Science and Technology	6	1,04%	92	18
Waste Management & Research	4.432 Q2 (107/279)	Environmental Sciences	26	4,51%	143	Azapagic, A	United Kingdom	University of Manchester	5	0,87%	156	60
Science of the Total Environment	10.753 Q1 (26/279)	Environmental Sciences	23	3,99%	474	Zorpas, A. A	Cyprus	Open University of Cyprus	5	0,87%	67	31
Journal of Environmental Management	8.910 Q1 (34/279)	Environmental Sciences	14	2,43%	269	Ragazzi, M	Italy	University of Trento	4	0,69%	106	34
Environmental Science and Pollution Research	5.190 Q2 (87/279)	Environmental Sciences	13	2,26%	121	Lu, W	China	University of Hong Kong	4	0,69%	101	33
Journal of Industrial Ecology	7.202 Q1 (49/279)	Environmental Sciences	12	2,08%	536	Bao, Z	China	University of Hong Kong	4	0,69%	101	7
ACS Sustainable Chemistry & Engineering	9.224 Q1 (13/142)	Engineering, chemical	10	1,74%	79	Irabien, A	Spain	University of Cantabria	4	0,69%	73	51

*R* ranking, *N* number of documents, *%* from the total sample of documents (N = 576), *TC* total number of citations

The most influential authors are sorted by number of documents published and total citations, indicating the institutions and country which they work in, and the h-index –impact and productivity measure-. The most prolific author is Navarro Ferronato from the University of Insubria in Italy with 9 papers published and a total of 129 cites, followed by Vicenzo Torreta (8, 129) from the same institution. The prevalence of Italian researchers is in line with the country's overall recycling rate for all types of waste which reaches 68%, well above the EU average (57%) published in the “Third Report on the Italian circular economy in 2021” (ENEA 2021). Additionally, in 2020 several legislative decrees came into force that facilitated the implementation of EU directives on waste and the circular economy.

Institutions include the University of Hong Kong whose role in integrated and sustainable waste management is significant both at the research level (Hossain et al. 2021) and practical level in running the campus and encouraging waste reduction and recycling among all stakeholders (The University of Hong Kong 2021).

## Research trend topics in the field

### Co-occurrence analysis by VOSviewer software

Co-occurrence analyses the most frequent keywords in a research field regarding their jointly mention, represented by clusters (Callon et al. 1983). This method is widely used to identify research trend topics about a particular subject area according to the keyword frequency (Donthu et al. 2021). The closer two items are from each other, the higher the connection. Accordingly, those keywords with a higher association appear closer.

This analysis used the full counting network technique which points the total number of occurrences a concept appears in all documents. The normalisation parameter method with association strength was performed by VOSviewer, to normalise the link strength between keywords (Van Eck and Waltman 2010).

**Fig. 3:**
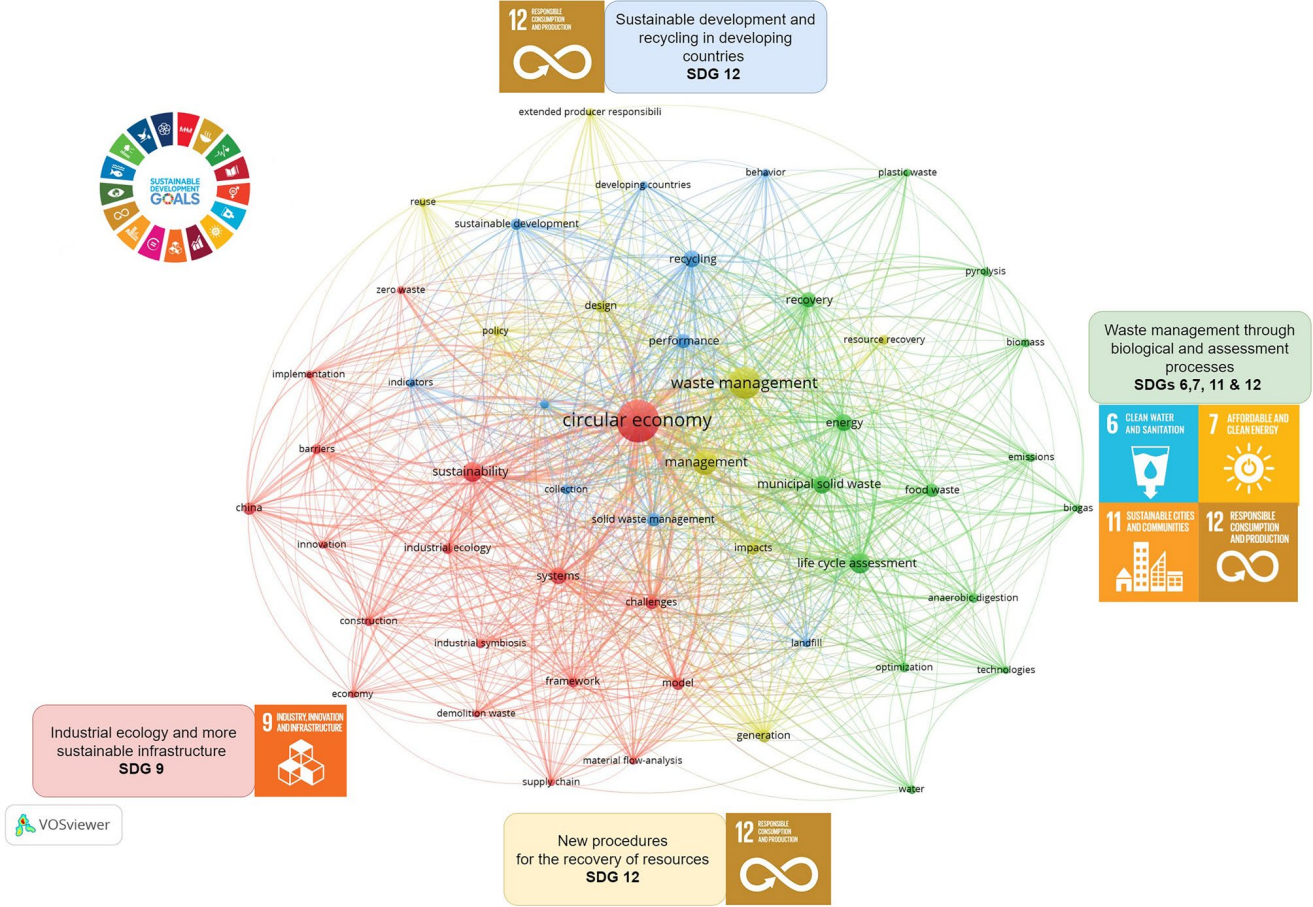
Co-occurrence analysis of keywords by vosviewer

Performing the analysis, different occurrence thresholds have been used to observe the network structure. VOSviewer software permits to perform a data cleaning to visualise a map created by text data merging terms using a thesaurus file (Van Eck and Waltman 2010). In our co-occurrence analysis we created a thesaurus to merge different keywords referring to the same item, such as ‘LCA’ and ‘life cycle assessment’, or ‘municipal solid waste’ and ‘municipal-solid waste’. Finally, a minimum of 13 occurrences of a keyword has been chosen from 2.868 words. 41 keywords met the threshold that represents the main items of each cluster. The keywords are divided up into main four groups of clusters coloured in red, green, blue and yellow in Fig. 3. The red cluster named ‘Industrial ecology and more sustainable infrastructure’ -SDG 9- focuses on the circular economy and industrial ecology with the aim of making industrial buildings and construction and demolition waste more sustainable, and on the challenges and barriers posed by these new models. The green cluster ‘Waste management through biological and assessment processes’ -SDGs 6, 7, 11 and 12- links the food waste and municipal solid waste and how anaerobic digestion and biogas can achieve a reduction in the use of energy and low emissions. Water treatment is associated with optimisation through new technologies. These studies use the life cycle assessment as a main tool for measurement. Sustainable development and recycling, considering indicators and behaviors in developing countries are shown in the blue cluster named ‘Sustainable development and recycling in developing countries’ -SDG 12-. Finally, the cluster in yellow studies the need to establish new policies and designs that would allow for improved waste management through resource recovery, such as the extension of producer responsibility beyond the sale of the product or service. It is therefore titled ‘New procedures for the recovery of resources’ -SDG 12-.

### Strategic diagrams and motor themes by SciMat software

Science mapping analysis displays how items from a particular field are linked to each other, determining the evolution and cognitive structure (Small 1999). In this study, keywords are the items used. The bibliometric mapping tool used to show the strategic diagrams is SciMat software. From the set of documents, it generates a knowledge base, in this case, the relationships between keywords are stored following a co-occurrence analysis. SciMat software grouped by plural to find similar items during the de-duplicating process (Cobo et al. 2012). For instance, keywords such as system and systems.

**Fig. 4:**
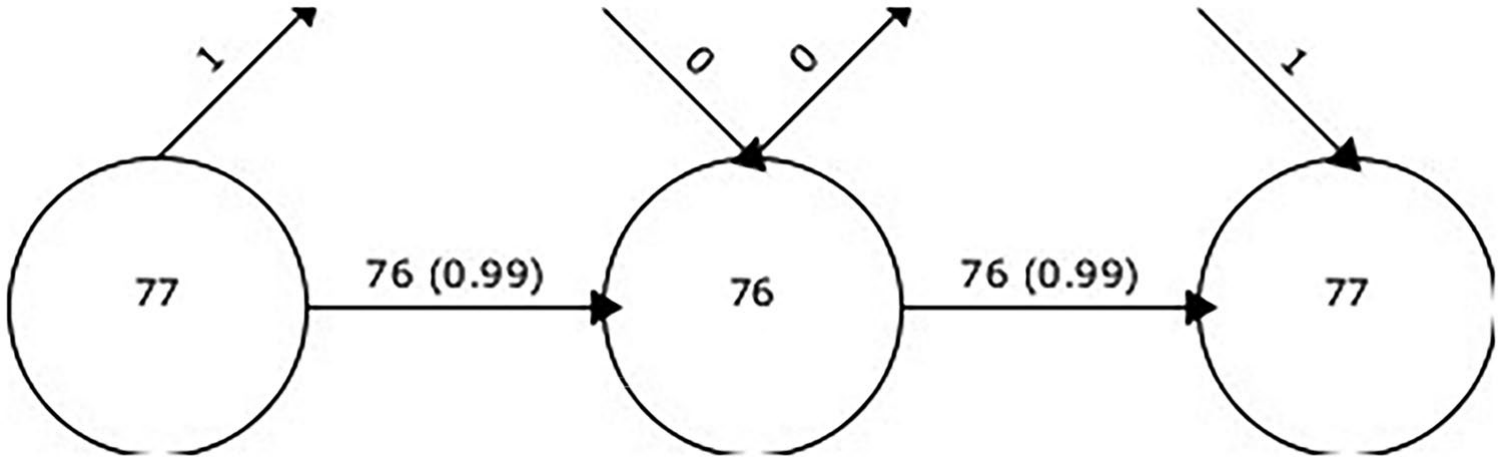
Overlapping map. Periods 1, 2 and 3 by scimat software

SciMat tracks a longitudinal framework that analyses the conceptual and intellectual evolution of a field. The normalisation measure chosen was the equivalence index. And to obtain the scientific map and the associated clusters and subnets, the clustering algorithm method followed was simple centers algorithm. The analysis is performed dividing the sample into three periods: period 1 with a total of 214 articles of year 2009 up to year 2019, period 2 with 155 articles of the year 2020, and period 3 with 189 articles of the year 2021. From a sample of 2,819 words, a total of 77 words have been considered, selecting only keywords with a minimum of 10 associated documents. As can be seen in Fig. 4, the stability index (0.99 and 0.99) indicates that there is a balance between the number of words from one period to the next.

The evolution map shows the results of the longitudinal analysis. The thick lines show the clusters that share a main theme, and the dashed lines are those that share themes other than the main theme (Cobo et al. 2012). In the first period the motor theme is circular economy, while in the second period the focus is on municipal solid waste.

**Fig. 5:**
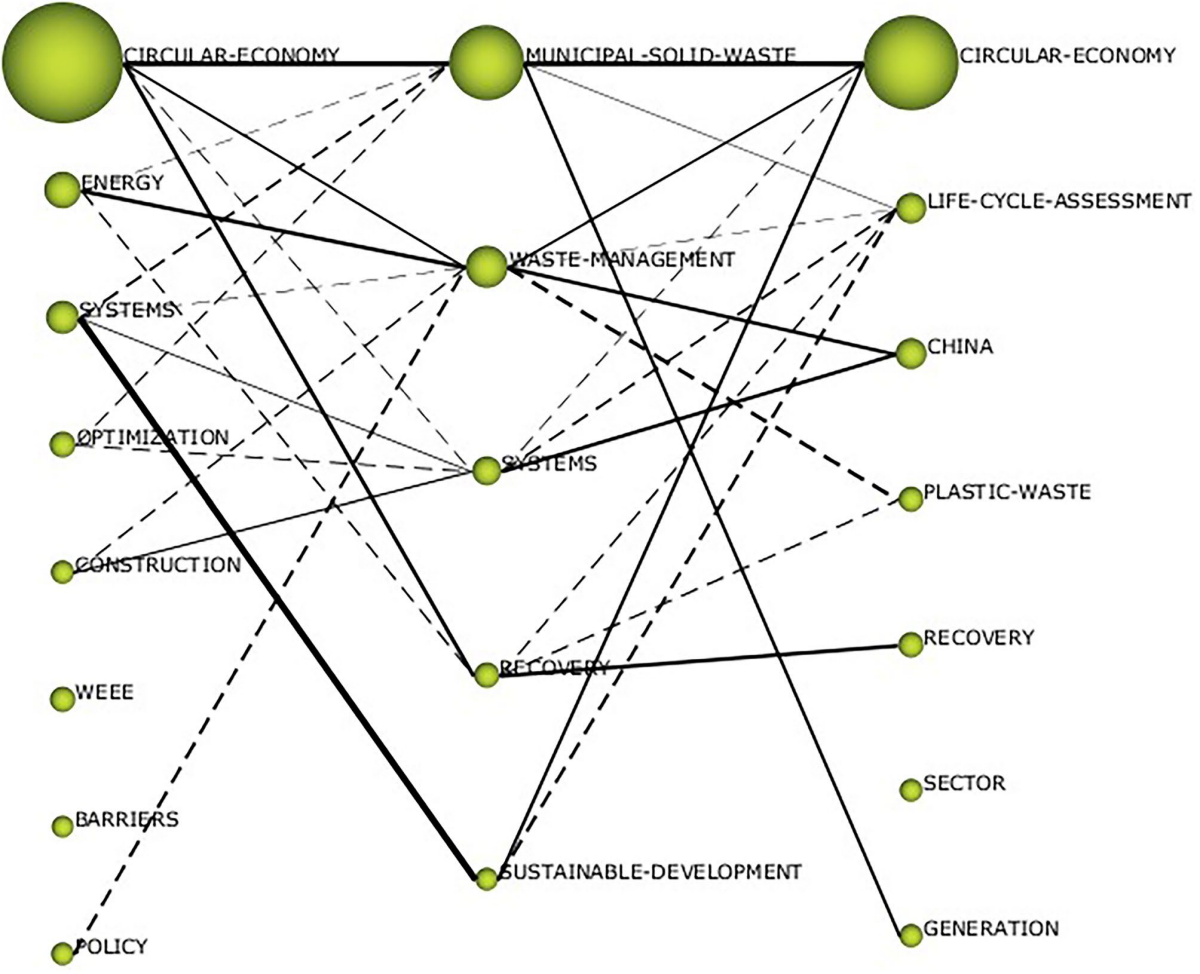
Evolution map. Periods 1 and 2 by scimat software

Figure 5 shows the difference between periods 1 and 2, from the more general to the more specific, with municipal solid waste oriented towards sustainable development -SDG 11-. In the third period focus returns to circular economy, with more dispersion apparent than in period 2, yet more specificity, as the number of clusters expands again. The massive generation of plastic waste generated during COVID-19 (Khoo et al. 2021; Vanapalli et al. 2021) could explain the interest in municipal solid waste management during period 2 and the emergence of concepts with plastics management in period 3. As a result, an evolution from the first period can be observed, with a strong focus on the implementation of circular economy and energy generation towards a circular economy centered on municipal solid waste.

**Fig. 6:**
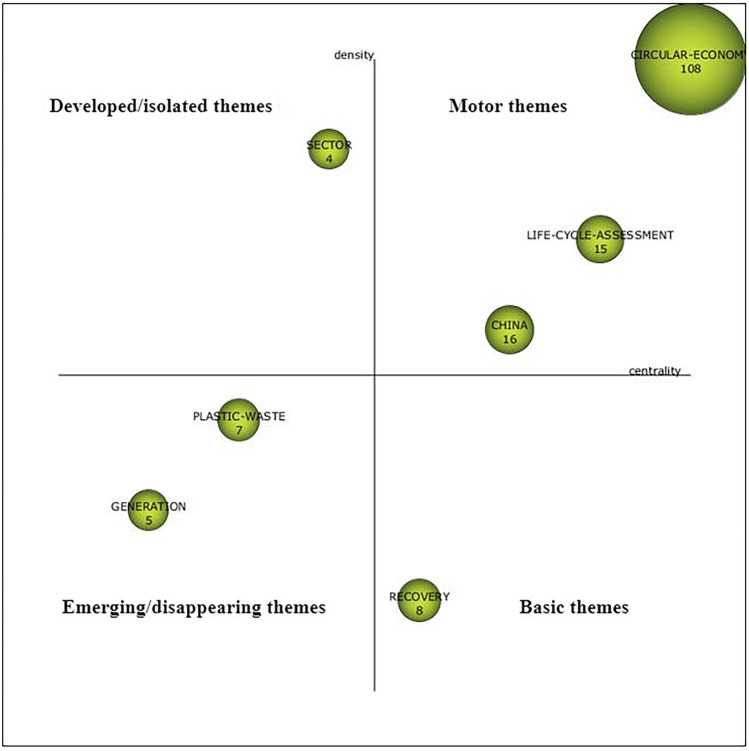
Strategic diagram. Period 3 (2021) by scimat software

This analysis is focused on the third period to gain better attention about the recent evolution of this field. Figure 6 shows a strategic diagram of Period 3 (year 2021) with four quadrants of the main thematic nodes according to the co-word analysis performed by SciMat. The strategic diagram displays the motor themes: ‘circular economy’, ‘life cycle assessment’ and ‘China’, developed thereafter, the basic themes: ‘recovery’ as a very specific and underdeveloped topic, it suggests a strategy towards circularity that is beginning to be considered, because many policies were only focused on promoting recycling (Ghisellini et al. 2016). The emerging or disappearing themes: ‘generation’, an emerging theme related to e-waste which is working on the reuse of products -SDG 12-, but circular economy is not applied in-depth. Regarding sustainable development and waste management, the environmental impacts are still a very large gap in the literature; ‘plastic waste’ is an emerging theme for circular economy, and it is studied within the pyrolysis and recycling process and new designs to improve the circularity -SDG 9 and 12-. ‘Sector’ appears as an isolated theme from circular economy, the literature is very cohesive in density due to its links with waste management case studies in different industries -SDG 9-.

Based on Fig. 6 ‘circular economy’, ‘China’ and ‘life cycle assessment’ appear as motor themes. These keywords present high density and centrality, thus they have been intensively and highly studied in literature. Which is why the following analysis is focused on them. ‘Circular economy’ is linked with ‘sustainability’ and ‘sustainable development’ according to the origin of circularity (Ghisellini et al. 2016). Likewise, the keyword ‘recycling’ relates to circular economy as a part of 3Rs principles, due to circular policies and their focus on recycling practices and strategies rather than other options -SDG 12-. ‘Municipal solid waste’ and ‘management’ is one of the most developed topics in the studies analysed and published during 2021 towards circular economy -SDG 11-.

‘China’ is a pioneering country in the implementation of circular economy policies, and strategies based on sustainability (Lieder and Rashid 2016). From a broad CE perspective, the country has incorporated these schemes due to the country’s rapid industrialisation and its growing efforts in research (McDowall et al. 2017). Indeed, the country is the largest producer of municipal solid waste (Wang et al. 2021) increased by COVID-19 (Vanapalli et al. 2021) and given its large industrial sector. The country is developing research that allows it to establish symbiotic relationships, to find new ways of using resources or converting waste into energy -SDG 7, 9 and 11-. It would be framed within the so-called industrial symbiosis, defined as the process by which waste from one industry or industrial process is converted into raw material for another (Provin et al. 2021).

‘Life cycle assessment’ appears far removed from circular economy, focusing more on waste demolition and construction management (Ahmed and Zhang 2021; Lu et al. 2021) -SDG 9-, and on plastic waste generation (Hossain et al. 2021; Pincelli et al. 2021).

## Review analysis

A systematic literature review was performed, considering the core documents with highest impact –those that appear at a minimum two nodes (Cobo et al. 2012)- from SciMat report. Selecting those articles from the three clusters that are presented as motor themes for period 3 (year 2021): ‘circular economy’, ‘China’ and ‘life cycle assessment’. Firstly, it was considered those papers with at least one citation (N = 51). Secondly, an in-depth analysis of those articles was carried out, compiling findings and future research lines of the 20 leading articles by number of citations (Table 2) according to the SciMat core documents.

**Table 2:** Findings and future research lines of the main articles related with circular economy and waste management during year 2021

**TC**	**Reference**	**Journal**	**Article category**	**Findings**	**Future research lines**
72	Vanapalli et al. (2021)	Science of the Total Environment	Theoretical	Actions and recommendations to reduce plastic waste related with Covid-19 designing policies, new technologies and products innovation (circular products), improving environmental behaviour, local production and consumption, incentives to recycling and efficiency	Extend the analysis with available data (empirical). Replicate the analysis to a post-pandemic scenario
16	Jeswani et al. (2021)	Science of the Total Environment	Empirical	Life Cycle Assessment shows that climate change impacts of chemical recycling and the production of circular plastics by pyrolysis are lower than energy's recovery and fossil resources	Improve the sensitive analysis. Consider other geographical areas. Future use of technologies treatment of end-of-life is needed
14	Sommerville et al. (2021)	Resources Conservation and Recycling	Empirical (44 commercial recyclers)	The quantitative assessment reveals a lack of circular thinking for the batteries end-of-life. It is necessary to have more options in reuse and recycling, closing the loop; and policies incentives improving circular practices	Increased knowledge about the recycling process of some of the components, their recovery and follow-up are required
14	Salmenpera et al. (2021)	Journal of Cleaner Production	Empirical (case study in Finland)	Economy, technology, culture and legislation solutions are considered for coordinate actions to identify critical factors in the promotion of circularity focusing on developers and intermediaries	Extend the analysis to other geographical areas and industries
11	Loizia et al. (2021)	Science of the Total Environment	Empirical (Municipal Solid Waste in Cyprus)	The study provides key performed indicators toward circularity and sustainable development goals, showing that more effectively citizens' participation in waste strategies, such as awareness activities is required	Extend the analysis to other geographical areas
10	Vardopoulos, et al. (2021)	Environmental Sciences and Pollution Research	Theoretical	Creating urban sustainable indicators of the environmental impacts from human activities, providing the correct strategy (DPSIR model) for optimizing MSW management effectiveness and efficiency in Greek	Lack of MSW generation data collection for comparison in the long-term. A need to amplify the indicators
9	Abou Taleb and Al Farooque (2021)	Journal of Cleaner Production	Empirical (waste recycling in Egypt, 27 councils)	Providing a model in municipal waste recyclable management from an accounting approach, with the highest circular economy gains and the lowest costs (cost-effective). Results show that developing countries must improve their circular and sustainable practices	Extend the data sample and periods for generalize the results. Apply to other industries
9	Massaro et al. (2021)	Business Strategy and the Environment	Theoretical	Improving circularity towards industrial waste management, focused on smart services. And how Industry 4.0 can be integrated in waste management: optimization software, robots, mobile applications	Considering more case studies is required and analyse separately the professional and scientific issues
9	Kazancoglu et al. (2021)	Business Strategy and the Environment	Empirical (Case study textile firm in Turkey)	The most important circular barriers are the lack of requirements and responsibilities for suppliers or manufacturers, and support from the government. Furthermore, one of the most fundamental factors is recycling policies for waste management	Differences of applying the model in other sectors. Extend the study to other geographical areas. The complementary use of different decision-making models is required, also considering other barriers
7	Di Foggia and Beccarello (2021)	Sustainable Production and Consumption	Empirical (Case study in 4.732 municipalities in Italy)	The use of landfill could be reduced by increasing waste-to-energy conversion. The study provides ideas for more efficient waste management with the use of new technologies	Comparative cost-effectiveness is necessary in future studies and extend the model to other geographical areas
7	Wu et al. (2021)	Sustainable Production and Consumption	Theoretical	Developing collective network-based bricolage process and adaptive institutional governance is an effective strategy for establishing an industrial-level circular economy towards the transition	Verifying the process in other geographical areas
7	Lombardi et al. (2021)	Journal of Cleaner Production	Empirical (Italian plastic packaging management)	Italian material flow analysis of the plastic packaging management and its circularity comparing the results with EU countries, showing positive rates on Italian recycling and energy recovery. The waste management efficiency must continue improving referring to its landfill levels	Calculate the eco-efficiency indicators and related material cycles. Limitations with the material flow analysis methodology such as the available data or the varying quality
6	Van Straten et al. (2021)	Sustainable Production and Consumption	Empirical (Case study 3 Dutch hospitals)	Showing the evaluating options of a hospital for calculating the save cost towards circularity: recycling the instruments, repairing for extending the life cycle of instruments, melting the steel into raw material and saving in handling waste costs	Extend the period under study (only 6 months are considered). A sensitive analysis for further understanding
6	Minoja and Romano (2021)	Journal of Cleaner Production	Theoretical	Studying Italian waste management and the TBL contribution to sustainability if its commitment is integrated from a managerial and governance process. Proactive participation of stakeholders is also fundamental for business models; and public firms are more suitable to sustainable issues	Ownership results are only replicable to other industries with the same institutional and legal circumstances. Further in-depth analysis of IC and sustainability is required. Extend the study to other geographical areas and industries
6	Sharma et al. (2021)	Business Strategy and the Environment	Theoretical	Investigating the prospects, impediments, and prerequisites in the transition to circular economy in SMEs in India conducting by a semi-structured interview. Financial issues, awareness, lack of experience and recycling subject are the main impediments. Prerequisites are related to innovation and motivation	Extending the sample under study for generalize the results. Applicate the analysis to other geographical areas
5	Jagodzinska et al. (2021)	Journal of Cleaner Production	Empirical (landfill case study in Belgium)	Studying close the loop with energy efficiency technologies towards circular economy by mining of existing landfills with the study of refuse-derived fuel of a waste excavated landfill in Belgium submitted to pyrolysis	Lack of data. The use of a more efficient technique of separation. Further analysis of the application is required
5	Elgie et al. (2021)	Resources, Conservation and Recycling	Empirical (Grenada case study)	Estimating the material flows waste stream of plastic, motor oil and tires for improving solid waste management towards circularity. This can be achieved by improving data collection, banning certain materials, applying the "polluter pays" principle, and developing a resource management plan for problematic materials	Lack of data. Extending the study to other geographical areas for further analysis
5	Woodard (2021)	Journal of Cleaner Production	Empirical (100 England SMEs)	Findings show the necessity of improving the efficiency of SMEs from England in waste management because of the use of household services to dispose of waste. Legislation, develop a holistic waste management system more effective, and increase the waste's awareness are key to achieve circularity	Comparison with other geographical areas. More in-depth review of local authorities
5	Foschi et al. (2021)	Environmental Science and Pollution Research	Empirical (Emilia Romagna región case study)	Promoting consumer's awareness, eco-design, a deposit-refund system, reduction of plastic waste, investing in a new industrial infrastructure of recycling, and the support to remanufacturers are the main recommendations of the work	Stakeholders’ participation is required and extend the analysis to other geographical areas
4	Khoo et al. (2021)	Journal of Hazardous Materials	Theoretical	Recommendation and future prospect and challenges in plastic waste management highlighting: increase awareness, policies, incentives and regulations, production with recycling purposes, new technologies for packaging,	More in-depth analysis about plastic waste during and post-COVID19 pandemic. Applicate to a real case of study

*TC* total number of citations

Citation analysis is a measurement widely used that considers a paper highly cited as relevant in a field (Zupic and Cater 2014), enabling us to evaluate the influence of a research topic. Also is used as a tool to detect emerging and research trends (Chen 2006).

Municipal Solid Waste (MSW) -SDG 11- is one of the main topics. Many of the papers related are case studies such as Vardopoulos et al. (2021) which developed a Driver-Pressure-State-Impact-Response (DPSIR) model to evaluate and assess the Municipal Solid Waste practices in Greek municipalities. Abou Taleb and Al Farooque (2021) concentrate on full cost accounting in 27 Egypt councils designing pricing model ‘Pay-As-You-Throw (PAYT)’ for municipal waste recycling. Wielgosinski et al. (2021) performed an analysis of the Polish municipal solid waste management through a balance model for assessing the impact of increasing the level of recycling, whilst Istrate et al. (2021) studied the municipal solid waste management in Madrid with a material flow analysis. Similarly, Tong et al. (2021) analyses the solid waste management system and the cause-effect relationship of households in Vietnam. Di Foggia and Beccarello (2021) highlighted the fact that the waste management chain in Italy focuses on waste-to-energy plants, calculating market measures towards circularity. In addition, in the region of Brescia, Italy, Bertanza et al. (2021) examined the evolution of municipal solid waste evolution with mass flow analysis of medium firms. Solid waste management in Brazilian universities is explored in the Nolasco et al. (2021) paper, which developed a qualitative-quantitative analysis, identifying factors of university campus waste management.

Plastic waste management is greatly studied in connection with circularity practices in many of the articles published during 2021, such as the case studies carried out by Foschi et al. (2021) on the Emilia Romagna plastic waste recycling system, following the European Commission Plastic Strategy. Similarly, Wu et al. (2021) outlines how Taiwan achieves circular economy in plastic waste from an industrial level, owing to collective bricolage. Some of the papers outline COVID-19 and the excessive use of plastics, coinciding with the most cited article of the sample (Vanapalli et al. 2021) which address COVID-19 plastic waste generation and the use of more sustainable technologies. The Khoo et al. (2021) paper provides recommendations for adopting effective plastic waste management due to excessive use during the COVID-19 pandemic. Pikon et al. (2021) shows the influence of COVID-19 on waste management from an economic impact perspective, highlighting the changes in municipal solid waste during the pandemic in the Polish market. Furthermore, increasing attention is being paid to biodegradable plastics as an alternative to conventional plastics. Ghosh and Jones (2021) examine upcoming trends, potential future scenarios, and the material value chain perspective of biodegradable plastics, whilst Kakadellis et al. (2021) categorizes qualitative data about biodegradable plastic strategies in United Kingdom -SDG 12-.

In the studies examined, the management of food waste is also analysed -SDG 11 and 12.- Zarba et al. (2021) analyses the Italian agri-food effectiveness towards circular economy regulatory; Provin et al. (2021) examines the reuse of food industry waste for the manufacture of biotextiles in the framework of the circular economy and the SDGs. This inter-industry collaboration would be part of the industrial symbiosis referred to above -SDG 9-.

In a similar vein, and related to SDG 9, the last process analysed by the most cited studies is the pyrolysis process, which allows thermal degradation of waste, associated with landfill mining, extracting valuable materials from the remains of materials deposited in landfills (Jagodzinska et al. 2021). Martínez (2021) discusses the opportunities and challenges of pyrolysis in Latin America.

## Discussion

This section is based on the results obtained from the bibliometric clusterisation, and the review of the 20 most cited articles. The number of articles published in the field have increased since 2015, corresponding to the United Nations Agenda 2030 and the 17 Sustainable Development Goals focused on improving and achieving education, health, economic growth and reducing inequality, as well as preserving forests and oceans (United Nations 2015). It is also remarkable to note the growth between years 2019 and 2021 due to new strategies and worldwide circular policies implemented in the field of waste management, such as the ‘Circular Economy Action Plan for a greener and more competitive Europe’ based on the prevention of waste and seeking improved local waste and raw material management (EU 2020; Camana et al. 2021). Although the "Agenda 2030" or "SDG" themes were not found in any of the clusters, the rest of the themes are closely related to their fulfilment. Moreover, circular waste management not only contributes to several SDGs, but also creates synergies between the goals.

A significant trend in the literature has focused on waste recycling (SDG 11 and 12), which is essential, yet insufficient if sustainable production and consumption is to be achieved by 2030. The main research topics analysed in the articles published during year 2021 focus on (1) Municipal Solid Waste (MSW) with the design of new municipal waste recycling models such as the Pay-As-You-Throw (PAYT) pricing model (Abou Taleb and Al Farooque 2021), (2) the importance of plastic waste (Khoo et al. 2021) and its recovery as a tool in the implementation of circularity principles (Ferreira et al. 2021), increased by the generation of plastic waste during the COVID-19 pandemic (Khoo et al. 2021), and (3) the reduction of food waste or its application in bio-textiles (Provin et al. 2021) or as an energy source -SDG 9 and 11-.

Going one step further should be considered in achieving further targets of this goal. On the one hand, a reduction in waste generation and a search for more sustainable disposal options for waste that cannot be recycled are required, e.g., through new processes such as waste pyrolysis (Jagodzinska et al. 2021) -SDG 9-. On the other hand, extending the lifetime of products by finding additional, new uses for them, eliminating planned obsolescence or repairing the product at a cost lower than buying a new product (Ghisellini et al. 2016) -SDG 12. Complementarily, waste generated in one sector can be used as a raw material in another sector or as a source of energy in the case of organic waste -SDG 7 and 9-.

## Research agenda

**Fig. 7:**
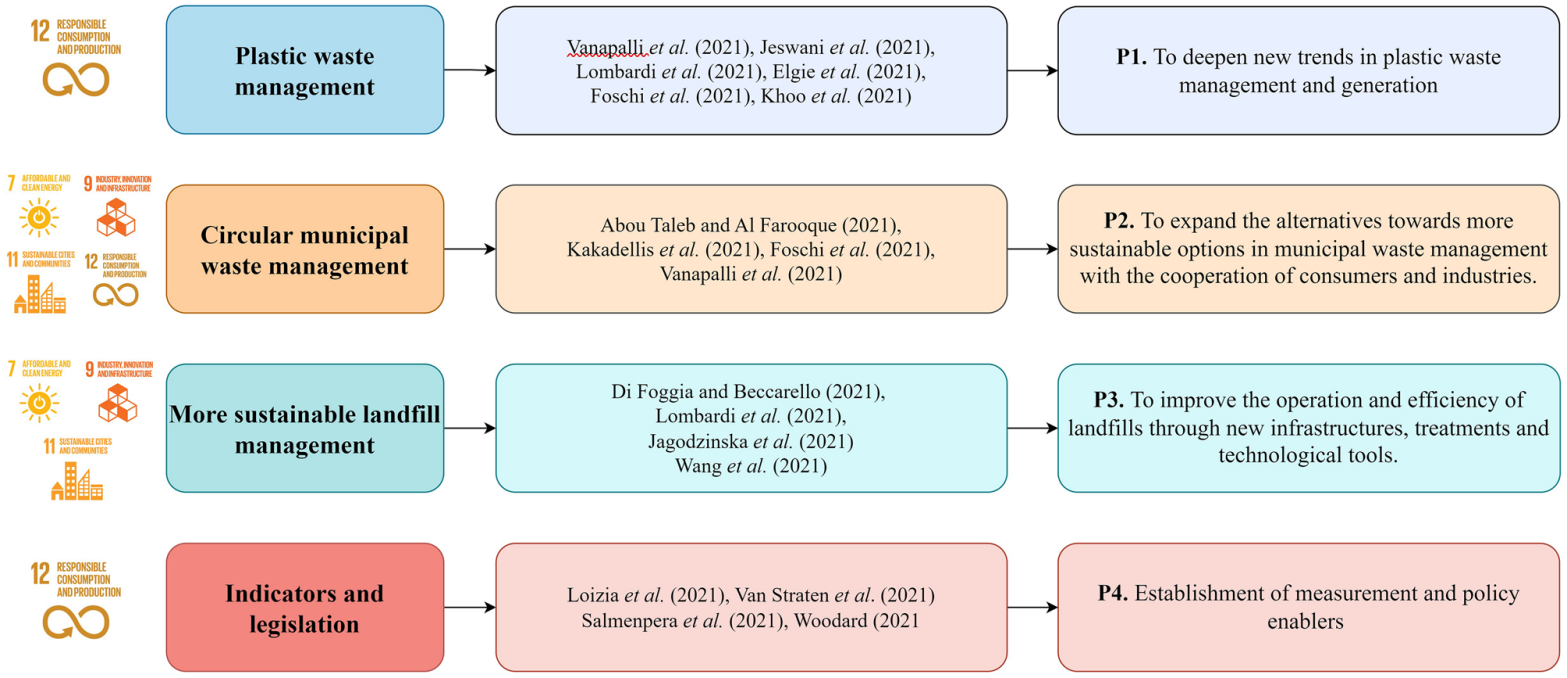
Research agenda propositions diagram

The research agenda provides guidance to scholars in future related-research directions. The agenda is based on the previous in-depth analysis of the 20 articles included in the review. Considering the analysis and the ensuing discussion, the following proposal is put forward for the circular management of waste management to accelerate the fulfilment of the 2030 Agenda. Moreover, this could fill gaps and give opportunities for further development. Figure 7 collects the research agenda propositions.

### New trends in plastic waste management and generation (SDG 12)

One of the most researched materials in the most cited papers is the use of plastic -6 of the 20 papers analyse this issue-. Firstly, because of the significant increase in waste associated with it after COVID-19 (Vanapalli et al. 2021; Khoo et al. 2021). Secondly, because of the need to progressively replace it with other materials such as biodegradable plastics, which implies the use of renewable raw materials. In short, solutions must be proposed to current plastic waste, the quantity of which threatens the habitat of numerous species, and measures must be taken to curb its expansion and offer alternatives in sustainable materials.

It is worth noting that no studies have been found that analyse the legislative challenges associated with the progressive elimination of plastic in products such as bags or single-use items.

**Proposition 1:** To deepen new trends in plastic waste management and generation.

### New pathways in the circular management of municipal waste (SDG 7, 9, 11 and 12)

The second line of the proposal relates to circular municipal waste management -SDG 11-, a topic of great interest in recent research (Abou Taleb and Al Farooque 2021), growing due to recent global crises. However, the approach that has analysed this topic focuses mainly on waste recycling.

A broader focus is needed, considering other alternatives such as the reduction of waste generation, reuse and the use of Organic Fraction of Municipal Solid Waste (OFMSW) as a raw material or energy source in other sectors. Compared to incineration, which is highly polluting if the organic waste is mixed with other types of waste, there are more sustainable and energy-efficient alternatives such as anaerobic digestion (Kakadellis et al. 2021) -SDG 7-. This requires consumer awareness and training –SDG 12- in waste separation, adequate facilities for the process and greater cooperation between industries (Foschi et al. 2021; Vanapalli et al. 2021) For the latter option, it is recommended that tools such as industrial symbiosis be explored in greater depth -SDG 9-.

**Proposition 2:** To expand the alternatives towards more sustainable options in municipal waste management with the cooperation of consumers and industries.

### Towards more sustainable landfill management (SDG 7, 9 and 11)

In contrast to traditional landfill management, new infrastructures, treatments and smart technologies are proposed to improve recycling and waste disposal. Among them, (1) the construction of waste-to-energy plants makes it possible to burn solid waste to power electricity generators (Di Foggia and Beccarello 2021) –SDG 7-; (2) pyrolysis process for thermal degradation of waste, reducing waste accumulation (Jagodzinska et al. 2021) –SDG 11- or (3) Industry 4.0 can be applied in waste treatment -SDG 9- for more efficient technique of separation models in waste management addressing circular economy practices (Wang et al. 2021). This line of research has a profound relationship with municipal waste management, given the importance of municipal waste in current landfills.

**Proposition 3:** To improve the operation and efficiency of landfills through new infrastructures, treatments and technological tools.

### Establishment of enablers in the implementation of circularity: Design of indicators and development of legislation (SDG 12)

Optimising waste management processes requires the establishment of measurement indicators. These indicators should be of a different nature and go beyond the economic or environmental quantification of targets. They should include social aspects such as awareness raising (Loizia et al. 2021; Van Straten et al. 2021). Additionally, along with technological and economic tools, the creation of a legislative framework is a critical factor in the implementation of circularity in waste management operations (Salmenpera et al. 2021; Woodard 2021).

**Proposition 4:** Establishment of measurement and policy enablers.

## Conclusions

Circular waste management focuses on reducing the amount of waste generated and reintroducing the waste, once treated, as new material or energy in production, keeping the material in a cyclical flow within the same or another sector (Demirbas 2011; Salmenpera et al. 2021). It, therefore, implies reaching a new level of treatment, complementing the recycling option with a holistic view of the problem. The application of circularity principles in waste management can contribute significantly to the fulfilment of the 2030 Agenda, as it impacts several of the SDGs -6, 7, 9, 11 and 12-.

According to the research questions presented, the scientific literature structure of the field of waste management and circular economy (RQ1) has been analysed, showing that the most productive sources come from the field of environmental sciences, which conditions the main topics investigated and shows a clear lack of attention to social sciences. The most prolific authors come from two countries with a strong interest in environmental research in general and waste management in particular—Italy and China. In the case of China, this is due to its strong productive fabric and a prominent role in the generation of waste from the COVID-19 pandemic.

Concerning RQ2, four clusters have been obtained related to industrial ecology -SDG 9-, waste management from the application of bio-based processes -SDGs 6, 7, 11 and 12-, water treatment, sustainable development and recycling in developing countries -SDG 12- and the cluster on new procedures for the recovery of resources -SDG 12-.

To conduct analysis of the central topics and the patterns we used SciMat software, dividing the articles published in the field into three periods (2009–2019, 2020 and 2021) showing the scientific literature development, as can be seen in the evolution map (Fig. 5). The motor themes showed in the strategic diagram of the third period are circular economy, life cycle assessment and China; recovery is a basic theme; the emerging themes are generation and plastic waste; and sector is a developed theme. Referring to RQ3, the results provided from the systematic literature review are in line with the central topics pointed out previously. Many of the studies published during 2021 pertain to motor themes circular economy and China, and to plastic waste as an emerging theme.

The most cited articles and the previous bibliometric analysis have shown the great interest generated among scientists in the management of urban waste and plastic waste, which has increased in the last two years in relation to sanitary waste. The circular economy means that recycling is not enough in the management of this waste. In addition to the reduction in the generation of waste, the incorporation of the "bio" concept in its treatment, which allows fibres, bioplastics and other biomaterials to be obtained, has been added. Along the same lines, the treatment of food waste allows it to be converted into animal feed, biofuels or even textiles. However, among the most cited articles, no research related to the use and recycling of wastewater was found -SDG 6-. Further research is needed to enable its use for biomass production or as a source of nutrients for micro-organisms of interest (Kaszycki et al. 2021).

The establishment of three research propositions completes this research (RQ4). In this way, it is crucial to develop three fundamental aspects. First, the use of new technologies to meet the various needs raised. Secondly, a new approach to urban waste management is required. And thirdly, to develop research from a holistic perspective that will require the use of theories and sciences from the environmental, social and economic fields.

### Theoretical contributions

The results of this study offer academic contributions about circular waste management. Among the theoretical contributions is the establishment of state-of-the-art research on waste management linked to the circular economy, which will guide future research and fill existing gaps. To offer the most complete research review possible, a mixed methodology—bibliometric and systematic review of the most cited recent research—has been used. A bibliometric analysis was carried out with two software tools, taking advantage of the potential of both. Using complementary software validates the analysis results. In addition, this article provides a framework for research as a guiding point in waste management.

Thus, lack of social research is a major drawback that requires urgent incorporation of new social or multidisciplinary studies. It can be considered that social and economic issues have not been sufficiently addressed in the literature. None of the clusters obtained have these dimensions as their motor theme. Dropping SDGs such as 8 -decent work and economic growth-.

### Practical contributions

A guideline for practitioners about circular waste management is required. Findings reveal the need for a reference framework for scholars, practitioners and institutions.

This article implies practical contributions for governments to achieve a transition towards more circular waste management. The research shows the technical feasibility of substituting certain materials, mainly plastic, or applying techniques that allow a step beyond recycling. It is necessary to focus on actions based on recovery, reduction, remanufacturing and redesign of plastic waste to fill this gap (Olatayo et al. 2022). Highlight the policy spillover effect, which means that support for some public fees—for example, plastic bag fees—may imply greater support for other environmental policies related to waste reduction (Thomas et al. 2019). This could facilitate positive transitions towards environmental behavioural changes. In addition, public–private coordination is required in the implementation of new legislation (Foschi et al. 2021).

The significant "bio" trend has spread to different types of waste and sectors. Thus, the circular management of waste will require the development of infrastructures, technologies and processes oriented to its application, which means waste management with less environmental impact, but also a generation of value of the product derived from the waste. This value can be manifested in new products -whether or not related to the original sector of the product from which the waste is derived- or renewable and sustainable energies (Ferreira et al. 2021; Kaszycki et al. 2021). For this, these processes require the establishment of cooperation tools between industries in such a way that we can establish symbiosis between them (Provin et al. 2021).

### Limitations and future research lines

Addressing the limitations of this study, it’s worth underscoring the fact that WoS was the exclusive Database used to retrieve the final sample under analysis, and only articles published in English are studied, other languages were not considered. Despite the use of VOSviewer to display the co-occurrence analysis, the interpretation of the results is subjective, in accordance with the papers reviewed. In future works, other software can be combined such as CiteSpace or HistCite to visually create scientific maps.

Regarding future research lines, the following aspects are considered a research agenda in the field of waste management and circular economy. The need to incorporate into waste management from a circular perspective such as: circular bioeconomy models, the construction of more robust eco-efficiency indicators to improve measurement and comparison between regions, and the consideration of new processes and techniques in the management of urban, food and plastic waste. Research is also required to manage waste in the construction and demolition of buildings and infrastructures from a sustainably innovative standpoint.

The challenges facing waste management in meeting the 2030 Agenda are considerable. Circular economy facilitates the pathway but is not a miracle tool. The contribution of companies and industries requires the collaboration and awareness of consumers. To this end, public institutions must generate policies, regulations and incentives that create the most favorable framework possible. Having already surpassed half of the set timeframe towards meeting the SDG targets, urgent measures are required, and the Academy must lend its support in this regard.

## Data Availability

Data was retrieved from Web of Sciences database.
